# Coastal Zone Information Model: A comprehensive architecture for coastal digital twin by integrating data, models, and knowledge

**DOI:** 10.1016/j.fmre.2024.06.003

**Published:** 2024-06-22

**Authors:** Zhaoyuan Yu, Pei Du, Lin Yi, Wen Luo, Dongshuang Li, Binru Zhao, Longhui Li, Zhuo Zhang, Jun Zhang, Jiyi Zhang, Wenchao Ma, Changchun Huang, Shuo Li, Xiaolu Yan, Guonian Lv, Linwang Yuan

**Affiliations:** a School of Geography, Nanjing Normal University, Nanjing 210023, China; b Jiangsu Center for Collaborative Innovation in Geographical Information Resource Development and Application, Nanjing 210023, China; c Key Laboratory of Virtual Geographic Environment, Ministry of Education, Nanjing Normal University, Nanjing 210023, China; d College of Geography and Planning, Chengdu University of Technology, Chengdu 610059, China; e College of Marine Science and Engineering, Nanjing Normal University, Nanjing 210023, China; f School of Geographical Science, Nantong University, Nantong 226007, China; g Center for Studies of Marine Economy and Sustainable Development, Liaoning Normal University, Dalian 116029, China

**Keywords:** Coastal zone information model, Coastal digitization, Coastal knowledge cognition, Data and model integration, Coastal digital twin

## Abstract

The coastal zone represents a critical intersection of naturally ecological and socio-economic processes. The abundance of data, models, and knowledge derived from various sources in coastal zones facilitates us to integrate them to better understand the evolution of coastal environments. This paper proposes a comprehensive framework of Coastal Zone Information Model (CZIM) to integrate multi-domain coastal information. The core idea of CZIM is to integrate multi-discipline coastal data, models, and knowledge for standardized governance, so as to carry, express, and apply coastal information by the digital system approaching the coastal digital twin. The CZIM framework includes four aspects: coastal data governance, model integration, knowledge engineering, and system construction. We perform a detailed literature review to illustrate the demands and challenges related to those four. The components of each aspect and their interlinks are introduced subsequently, and the future challenges of constructing coastal digital twins relying on CZIM are discussed. CZIM aims to strengthen the ability to organize, manage and apply refined coastal information to support more efficient, scientific, and intelligent decision-making in response to gradually volatile forces from both human activities and natural events, now and in the future. This paper provides a valuable reference for the next generation of coastal digitization in the target of the coastal digital twin.

## 1 Introduction

The coastal zone extends from the coastline, encompassing both land and sea, serving as a crucial intersection between natural ecology and socio-economics. This zone is characterized as a dynamic system where natural changes and human activities are interconnected [1]. Recent reports highlight sustainability conflicts concerning the present and future of coastal zones, which encompass natural phenomena such as shoreline change [2], sedimentation and erosion [3], and water eutrophication [4]. Concurrently, human-induced pressures include urbanization [5], coastal reclamation [6], microplastic pollution [7], and disturbing ocean events [8]. Moreover, external forces intensified by global climate change, such as global warming, rising sea levels [9], and frequent typhoons [10], further exacerbate these complexities. These pressures expose significant vulnerabilities in the socio-economic and ecosystems of the coastal zone. Using primarily advanced digital tools for integrated digitization in coastal zones is essential to achieve sustainable development in response to these pressures.

Several publications have reviewed the challenges in coastal observations [11], models [12], and knowledge systems [[13], [14]]. Implementation of major projects such as new fundamental surveying and mapping in coastal observation has shifted the basic unit of digital coastal management from two dimensions to three dimensions. This improvement has significantly enhanced the accuracy of geometric scenes [15]. However, the standards for integrating coastal information from both land and sea into a unified three-dimensional scene are still insufficient. Regarding model analysis, the demand for refined management has driven the coastal analysis model towards multidisciplinary collaboration. Nevertheless, existing coastal analytic models primarily focus on representing the static distribution of natural factors and statistical information in a relatively single discipline [16], lacking the consideration of more informative results and constrains from other disciplines. For the knowledge part, coastal informatization still needs to establish an integrated knowledge system to integrate, apply, and interact with coastal-related data, mechanism processes, and management policies in different disciplines to make decisions more inclusive.

Advanced digital technologies provide innovative ideas for developing the new-generation coastal zone systems. Importantly, many coastal-related international programs, in a spirit of global collaboration, focus on digital twin technologies (digital twin can be understood as a digital equivalent of a real-world system that effectively combines observations, artificial intelligence (AI), advanced modeling, and high-performance computing to unite digital replicas, forecasting, and what-if scenario simulations), such as Destination Earth (DestinE) from the European Union [17], Decade of Ocean Science for Sustainable Development from the United Nations [18], and Transparent Ocean from China [19]. Intelligent interactions and expressions are expected to be developed at the software system level to increase the cognoscibility and comprehensibility of coastal information. On the other hand, AI technologies provide many valuable applications in enhancing coastal-related data accuracy and substituting traditional numerical models [[20], [21]]. The state-of-art AI technologies, such as Physics Informed Neural Networks [22], Multimodal Deep Learning [23], and conversational Large Language Models (LLM) [24], are not only advantageous in improving data quality but also in exploring natural mechanisms [25] and integrating the multidisciplinary knowledge. This sparked the rethinking of the application of AI technology in coastal zone sciences. How to effectively coordinate the digital resources in the coastal zone to support future-oriented intelligent research is an imperative issue that must be triggered.

In our opinion, the coastal zone digital twin is a comprehensive project covering the process from the underlying data management logic to the top-level application architecture to realize information integration and guide the feedback between the real coast and the virtual coast. As a typical region for the management and application of big Earth data [26], a standardized framework for integrating coastal data, models, and knowledge is urgently required. However, such integration is challenging. The complexities of coastal management are deeply rooted in its cross-domain characteristics [1]. As data and models from various coastal-related domains have their own specific application scenarios, the expertise barriers between different coastal stakeholders make the application of knowledge in integrated coastal zone management complicated [27]. The presence of commonly used standards, such as independent land and ocean coordinate systems, makes integration particularly challenging. The intelligent and refined development of coastal management has been limited by the incomplete, controlled, and isolated concept of the division of land and sea for so long [[28], [29]]. A comprehensive framework is required to support the formulation, simulation, optimization, and interpretation of coastal zone systems to benefit the development of the coastal zone digital twin.

This paper aims to develop a coastal-specific oriented framework for information integration and application. The framework involves four perspectives: coastal-wide data governance, multi-domain model integration, standardized knowledge system, and scenario-based interactive information system. To analyze the prominent challenges faced by coastal digitization, we review the development of those four aspects. Based on this, a conceptual model of the Coastal Zone Information Model (CZIM) is proposed. The CZIM is designed to mitigate domain barriers in coastal information applications, share information with efficient standards, and apply information with intelligent and realistic interaction. Furthermore, we discuss the critical components of CZIM and the construction orientation around these objectives. Additionally, with the support of the China Nature Key Funding Program “Coastal Zone Information Model for Scenario-based Modeling and Sustainable Management”, we look forward to synergizing more disciplinary fields, emphasizing the collaborative nature of this endeavor, to contribute to constructing the coastal digital twins not only for virtual visualization but also for the development of intelligent and practical applications for coastal zone management.

## 2 Literature review

### 2.1 Coastal zone data governance

The coastal spatial boundary separates two different data category systems, land and sea. A large number of high-quality land and marine factor datasets based on mature data platforms are utilized in coastal research, including the coastal environmental factors from land topography, ecological environment, socio-economic, marine meteorology, hydrography, ocean dynamics, and so on [11,30]. Coastal-related remote sensing technology provides a large amount of basic environmental data. In the land part of the coastal zones, remote sensing technology provides many large-scale monitoring, classification, identification, and inversion data for their environmental factors, such as coastal morphological change monitoring [31], coastal wetlands extent [32], mangrove extent [33], and the coastal vegetation classification [34]. In the sea part of coastal zones, remote sensing technology provides a large amount of monitoring data for the nearshore environmental factors, such as red tides [35], water quality [36], shallow water bathymetry [37], as well as disaster elements of sea fog [38], storm surges [39], and so on. With the improvement of the spatiotemporal resolution of satellite images and the accuracy of AI algorithms, the quality of coastal data has been significantly improved in recent years, such as coastal aquaculture ponds datasets during 2016–2021 at 10 m resolution [40] and wetland vegetation classification datasets at 5 m resolution [41]. Additionally, new types of basic surveying and mapping data, 3D reality, and tensor spatial-temporal databases gradually extend from land observations to three-dimensional ocean observations. 3D-based scene data, such as mapping 4D products^1^ and panoramic scenes (such as laser point cloud and 3D entity reconstruction) [[42], [43]], are introduced to combine high-fidelity visualization in coastal systems with 3D reality to create a more realistic and intuitive system platform.

Observations from the Internet of Things, balloons, drift/profile buoys, deep gliders, and real-time mooring provide reliable first-hand coastal environment information in the estuary, intertidal, and subtidal areas. For example, coherent arrays of free-drifting buoys are employed to directly measure the kinematics of the nearshore surface, providing reliable data for the study of the nearshore wave kinematics and wave-breaking processes [44]. The non-dispersive infrared monitoring system is used to investigate the variation of greenhouse gas emissions from different plant species, which is of great importance for coastal refined carbon estimation [45]. In-situ observations of surface chlorophyll-*a* from the northern bay provide reliable long-time-series baseline datasets for remote sensing inversion over a wide area [46]. However, these coastal observation sensors’ spatial and temporal distribution is generally sparse due to the high costs of equipment and projects. It is challenging to gain a complete picture of evolution process in coastal zones with insufficient data support.

The same situation also appears in data related to coastal administration. Different administration data are managed by corresponding departments, such as population density [47], regional innovation index [48], and land use [49]. Although the accuracy of these data has improved significantly, there is still no practical way to integrate them in comprehensive coastal management, since the value, confidentiality, and the target audience of coastal administration data make it difficult to collect and share. “What, where, and when” the administration data is effective in the entire management causal chain in the coastal zone is indistinct. An overall design needs to be oriented, especially from the land and sea integration perspective.

AI technology provides a possible way to mitigate some limitations in refined data use in the coastal zone. AI technologies are advantageous in improving computational efficiency and accuracy, constructing causal correlations, and fusing multi-source data. They are extensively applied in coastal-related research to enhance, downscale, and assimilate the coastal environmental factor data. For example, the multilayer perceptron artificial neural network was employed to reduce the bias of the coastal Digital Elevation Model (DEM) [50]. The deep learning structure with convolutional neural networks and long short-term memory layers was used to construct simulation ensembles for the coastal surge levels [51]. The deep learning-based super-resolution model was employed to predict the high-resolution spatial estimation of spectral wave parameters from the global ocean wave model of Simulating Waves Nearshore (SWAN) to provide 16 times higher resolution results [52]. Multi-source data fusion has become a potential trend in AI-based data enhancement [53]. However, data in coastal areas are loosely organized, making it challenging to meet the requirements of AI methods in terms of the sample size, data variety and quality. The challenges are mainly reflected in the scarcity of high-quality observations, the difficulty of data integration from multi-domains, and the difficulty of filling the space adjacent to the boundary in the coastal zone. The complicated coastal data is expected to be managed in a more standardized way [54]. Variable or polygonal grid methods are expected to be applied to fit better the coastal zone’s topography and boundary morphology [55]. Data governance framework needs to be developed to harmonize the demands of intelligent computation for refined coastal zone management.

As a typical multi-interface exchange zone on the earth, the coastal zone presents a unique challenge in managing and applying big earth data [26]. This challenge is characterized by a co-existence of sparsity and abundance in coastal data, which are cross-interface and cross-disciplinary driven by land-sea-air- human interactions. From the perspective of data management, integrating large volumes of data has become a burden in coastal management-related research [56]. The diverse stakeholders and management departments, such as spatial planning, disaster prevention, estuary management, and wetland conservation, each have their unique data application requirements. Coastal zone data are not effectively applied in a unified data governance model [57]. However, the data category perspective poses a challenge as different disciplines have different data classification systems, which hampers the integration of factor data into the overall metadata system. Similarly, the data association perspective presents a hurdle as the data among different disciplines lacks interconnection, making it difficult to collaborate toward scenario-level system applications.

### 2.2 Coastal zone model integration

Different scales and types of coastal models in various coastal-related fields play a crucial role in information production, transformation, computation, and analysis. Coastal models extensively cover natural, human, and information domains. Over the past few decades, advances in the development of unstructured grids, model nesting, ensemble models, and model coupling [31] have rapidly improved computational power and the ability to assess the model performance of physical and biological processes in the coastal zone [58]. Several land and sea simulation and assessment models have been applied to analyze coastal-specific dynamic processes. These include the tidal models (such as TPX and FES [59]), wave models (such as WAVEWATCH III and SWAN [60]), hydrodynamic models (such as Finite Volume Coastal Ocean Model (FVCOM) [61], Regional Ocean Model System (ROMS) [62], and Delft3D [63]), ecological models (such as Integrated Valuation of Ecosystem Services and Tradeoffs (InVEST) [64] and United States National Estuarine Eutrophication Assessment (NEEA/ASSETS) [65]), land-based hydrological models (such as Soil and Water Assessment Tool (SWAT) [66]), and groundwater flow models (such as SEAWAT [67], and Modular Three-dimensional Groundwater Flow Model (MODFLOW) [68]). Coastal-related process models have also been rapidly developed. For example, the Coastal Storm Modeling System (CoSMoS) is designed to thoroughly assess future coastal flooding exposure by integrating sea level changes, dynamic water levels, and coastal line change [69]. These models provide practical tools for sustainable management of natural coastal systems and improvement of sustainable urban infrastructure, especially in the context of rapid coastal urbanization and global sea level rise.

Although coastal process models have become progressively abundant, the integration is more crucial in the coastal zone since multi-domain process interactions characterize this region. However, it is challenging to integrate processes from different coastal domain models. Some models’ process parameters are site-specific and highly dependent on resolving bathymetric, coastline, and forcing variability. The idealization and parameterization of uninterested external/internal processes consistently result in the simulation results of the coastal zone model in a diffuse state with diverse uncertainties [70]. It is also infeasible for the model to accept another related process by modifying its inherent idealized configurations. Thus, more componentized sub-process models of the coastal zone are expected, which could provide the possibility of multi-process simulations by combining those sub-processes.

Some efforts have been made in this aspect. For example, simplifying numerical modeling in coastal zones is proposed to mitigate the application complexity, providing streamlined physical descriptions of multi-layer mechanisms and facilitating rapid simulations [71]. Additionally, AI technology offers potential application values. For example, regarding coastal environmental factor forecast, the AI-based wind waves forecast system was proven effective in the short-term forecast with near real-time response [72]. In terms of scenario simulation, the combination of artificial neural network and cellular automata model was used to predict the coastal urban development boundary and perform the multi-scenario simulations [73]. However, it will be more accessible to integrate process models from different coastal domains to construct the practical AI model if there is a standard model integration architecture to describe multi-domain process methods, guide the correlation of different processes, and constrain the proliferation of ensemble models. More detailed coastal zone land, subtidal marine, near-surface atmospheric, and subsurface processes must be categorized and correlated to support synergistic multi-domain modeling and the construction of integrated process simulation environments. A more unified architecture for model inputs, including grid generation and boundary forcing files, would significantly reduce the overhead and expertise required to apply a new model to the problems.

### 2.3 Coastal zone knowledge engineering

Coastal zone knowledge is accumulated from information generated at different natural and social evolution stages. It can refer to as any valuable information in coastal-related research, scenarios, or applications. As the coastal zone involves multiple disciplines and stakeholders on both land and sea, it is of great importance to utilize diverse knowledge to manage the coastal zone. As early as the 1990s, Fabbri [74] expressed coastal knowledge as the linkages and interactions among different coastal factors to facilitate the understanding of cause-and-effect relationships. After that, the challenges of utilizing and integrating diverse knowledge in optimizing coastal management were emphasized from the perspective of land-sea integration, global-regional synergy, and scientific-indigenous fusion. For the land-sea integration, most studies regard the coastal line as the boundary and overlook connections of the whole land-sea system, resulting the whole picture of land-sea systems is not recognized from the systemic perspective of coastal knowledge [27]. From the perspective of regional synergy, potential conflicts exist between the global environment and regional policies in response to the external pressures of the coastal zone, resulting in the inconsistency of development goals for multi-sectoral collaboration in coastal management [75]. From the perspective of knowledge fusion, a gap between coastal indigenous knowledge and scientific knowledge of sustainable development is exhibited. The direct links between scientific knowledge and stakeholder practices are absent to support sustainable development [27]. In summary, integrating coastal zone knowledge is essential for systematically comprehending and managing coastal zones. Nevertheless, Hinkel and Klein emphasized the potential cost of study, application, and interpretation of different domain knowledge in constructing the comprehensive coastal assessment model [76]. The ambiguous and non-standard coastal knowledge in different domains impedes the collaboration of model developers and the interpretation of model users for efficient decision-making [76].

The importance of building an integrated and interdisciplinary knowledge system in coastal zones is being emphasized. Integrating knowledge from various scientific disciplines through combination, interpretation, and communication could add value to decision-making processes in management [77]. The systematic integration of coastal multi-source information could be guided by the application knowledge of data, model, and their associations, improving efficiency and the understanding of the interactions between different system processes [76]. With its practical application, the integrated coastal knowledge system is advocated to perform [78], particularly in response to the pressures brought by global climate change on broader societal ideologies, security, and management issues [77].

Efforts have been made to construct the coastal knowledge system, such as the knowledge graph used to express and store the knowledge of nearshore maritime safety [79] and the coastal typhoon disaster assessment system [80]. Bibliometric analysis has been used to summarize the main ideologies of beach litter [81] and coastal indigenous knowledge applications [82]. However, despite the effectiveness of advanced tools like knowledge graphs and the recently prevalent LLM in integrating domain knowledge [[83], [84]], constructing a comprehensive coastal zone knowledge system is still in the early stages. Knowledge barriers in the division of departments and conflicts among international, national, regional, and sectoral regulations need to be further eliminated. The rules of natural and human processes need to be clarified. The relationships of “data to data”, “data to model”, and “model to model” in coastal digitization are still unclear, hindering the research in various coastal zone scenarios, comprehensive management, policy simulations, and system development. Hence, there is a pressing need for a comprehensive knowledge system of data and model definitions, applications, interfaces, and linkages to support the coastal knowledge application by model developers, coastal zone researchers, policymakers, and non-professional applicators.

### 2.4 Coastal zone system construction

The primary objective of constructing a coastal information system is to facilitate the efficient integration, expression, application, interaction, and dissemination of coastal zone data, models, and knowledge. It serves as a tool for professionals engaged in coastal research and assists non-specialists in applying coastal-related information. Over the past decades, with the advancement of computer system technologies such as spatial databases (including SQL and NoSQL databases) [85], data service standardization and architecture (such as WebService, Microservices and Cloud Services) [86], international standards (such as Open Geospatial Consortium (OGC)), OGC-based spatial mapping services (2D and 3D tile and feature service) [87], and computer graphics technologies (such as OpenGL and WebGL) [88], the functionality of coastal information systems has evolved from initially factor data displaying and downloading to provide interactive operations.

Coastal information systems such as data management and ecological information systems [89] have provided great convenience for users to apply coastal information. With the support of efficient system development tools, the development of coastal information systems has evolved from the professional dilemma to accessing a broader interdisciplinary collaboration. Under this trend, some specialized service systems has been developed, such as the Digital Shoreline Analysis System (DSAS) for regional shoreline changes analysis [90], OPENCoastS for automatic generation of coastal forecast systems [91] to increase the applicability of coastal-specific analysis and modeling. Additionally, the information framework of big earth data [92] and its successful cases such as Deep-Time Digital Earth [93], WorldWind and Skyline Globe [94] provide valuable architectures for the construction of the coastal zone information system.

However, there are several challenges in constructing digital twin-orientated coastal systems. Firstly, multidimensional visualization methods for land-sea natural factors in 3D scenarios have yet to be widely implemented. Transient and single-layer data visualizations are the mainstream expression strategies in coastal information systems. This does not adapt to changes in scenario-based coastal processes, such as large-scale typhoon encroachment or coastal hydrological variability. Secondly, there still needs to be a standard framework for the storage, transmission, and expression of coastal 3D information like those provided by the City Information Model and Building Information Model [95]. This is critical because harmonized standards allow the data to move from being built, shared, and applied by individual professionals to being accessible to public users to create a sustainable community for coastal digital twin development. Furthermore, the coastal models are expected to integrate the coastal data and model in the 3D reality scenarios, providing a more stereoscopic interaction response for decision simulation or risk assessment.

### 2.5 The challenges summary

The multi-interface coupled environment in the coastal zone introduces complexity in data governance, model integration, knowledge engineering, and system construction. Despite considerable research and achievements in the coastal zone, there are still significant demands and challenges in integrating comprehensive coastal information to address dual pressures from climate change and human activities, which is crucial for constructing a digital twin for the coastal zone.

(1) The current state of coastal zone data, categorized as “land-sea-air-human”, is characterized by diversity and a scarcity of high-quality data. The spatial management of multi-departmental data near the coast is notably inadequate. The loosely organized and non-standardized governance of coastal zone data in terms of category, space, and association, poses a significant challenge in supporting the comprehensive diagnosis and attribution of coastal changes.

(2) Coastal zones, shaped by continuous tidal actions, form an interactive environment where dynamic and static elements coexist. However, various coastal zone models idealize the coupled changes in the coastal zone within specific subdomains, assuming static boundaries, substrate, grids, and statistical backgrounds. This leads to a limited understanding of the comprehensive coastal system, constrained by subdomain-specific idealizations.

(3) Coastal zones accommodate diverse space utilization and resources within limited space. Different stakeholders grasp and apply coastal information faces potential domain restrictions. Fragmented management by various departments hinders information sharing, and inherent knowledge barriers impede practical application.

(4) The current state of coastal information systems is far from ideal. They struggle to handle storing, transmitting, and expressing multidimensional coastal factors and processes, particularly in accurately representing the high-dimensional factors and processes involving the land, sea, and air and their interactions.

In summary, as a fusion of diverse natural and human factors within limited space, the coastal zone has the characteristics of coupled process relationships and dynamic-static interactions. The data governance in coastal zones faces the complexity of factor category relationships and spatial associations. Coastal model simulations exhibit partiality and incompleteness in capturing the complexities inherent in coastal zones. The coastal knowledge has yet to be integrated and functioning due to the knowledge barriers among different disciplines. The coastal system platform is expected to move from a 2D plane to 3D real scenes, supporting intelligent interactions. In the absence of standardized and comprehensive integration across these dimensions in the digital content of coastal zones, managers need help synthesizing information from diverse domains, impeding their ability to make well-informed decisions. The comprehensive governance and integration of coastal zone-wide data, multi-domain models, and multi-granular knowledge are expected. Thus, it can facilitate the development of intelligent interactions and the establishment of a coastal digital twin. In turn, it can support standardized information applications to respond to the sustainable development of the coastal zone in harmony with human and natural elements.

## 3 Coastal zone information model

### 3.1 Definition and connotations

The CZIM can be understood as a holistic framework that integrates coastal-related data, models, and management operations spanning the entire land-sea-air-human interaction region. This integration is facilitated by the comprehensive coastal knowledge system, with the coastal digital system serving as the platform for information expression and interaction.

**Fig. 1:**
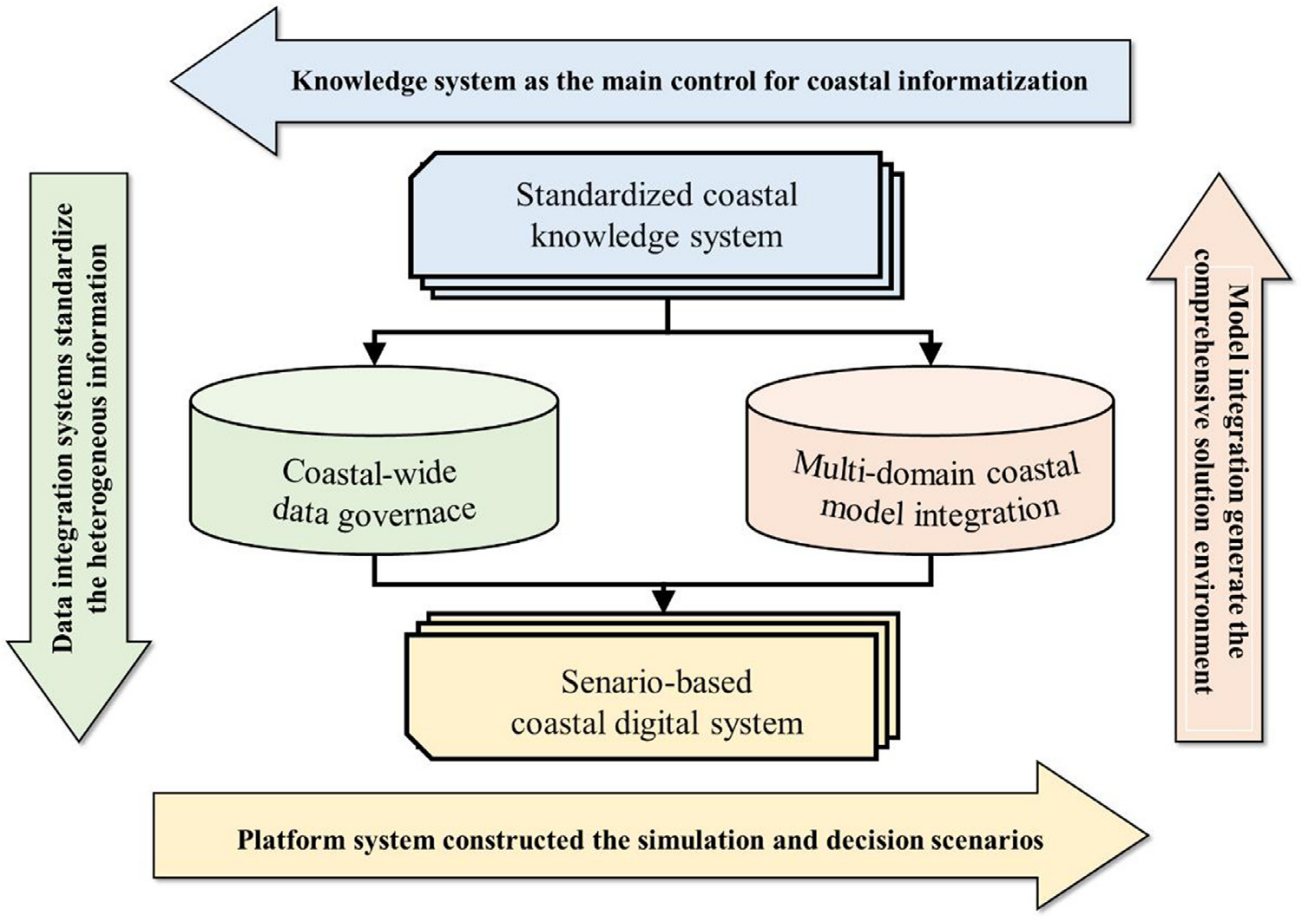
**Conceptual diagram of the CZIM**.

The main idea of the information model is to transform data into knowledge in an information application cycle, transforming the observed, simulated, and analyzed data into presentable, applicable, and interoperable knowledge. This includes the dynamic feedback between coastal reality and its digital systems, cooperation between coastal subsystems and integrated systems, and human-system interactions. To realize this iteration, the CZIM has four aspects of construction connotation: coastal-wide data governance, multi-domain model integration, standardized coastal knowledge system, and scenario-based coastal digital system (Fig. 1). Historical experience knowledge is leveraged to guide the comprehensive scenario design, and new information knowledge feedback complements and updates the knowledge pool. By continuously accumulating and updating, it is expected to develop broad collaborations across different coastal research fields and build a holistic approach to coastal integrated scenario in the CZIM’s entire construction and application life cycle.

### 3.2 The framework for the CZIM

**Fig. 2:**
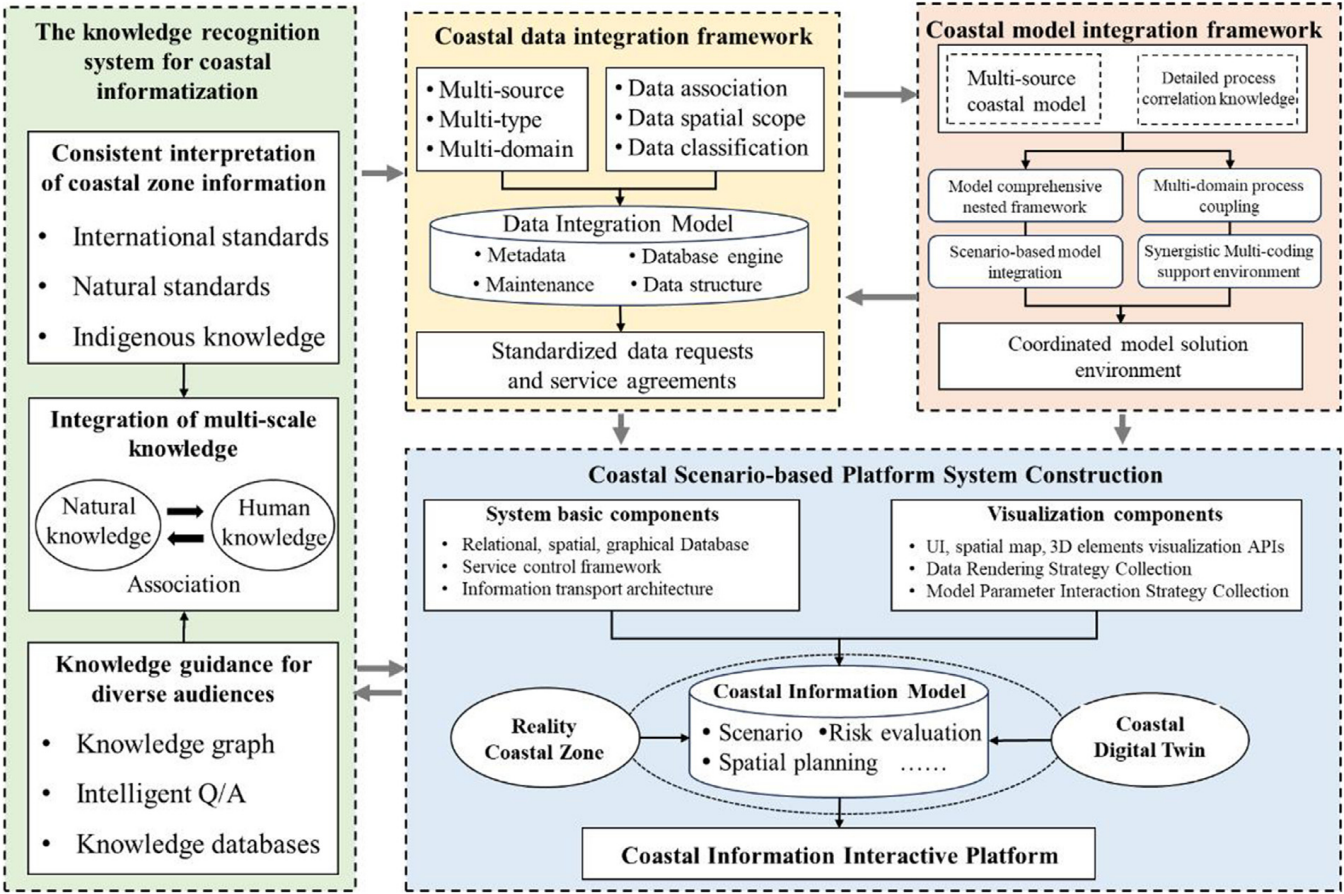
**The structural framework of the CZIM**.

The framework of CZIM covers coastal data and model integration, information system construction, and their transmission and collaboration. The architecture is shown in Fig. 2.

Coastal zone data governance encompasses three essential requirements: the unique classification system, spatial retrieval, and data linkage in different domains. These three requirements correspond to the essential features and problems in coastal data governance, which are the lack of data coupling across multi-interfaces, spatialization of the sparse data and the correlation of multi-domain data. Accordingly, three governance modules are essential. Firstly, the data classification system based on the hierarchical structure is necessary, which includes multi-interfaces (hydrosphere, biosphere, atmosphere, and anthroposphere), spaces (land, intertidal zones, subtidal marine, and surface, subsurface, and underground), and coastal management domains. Secondly, spatial data governance requires a polygonal variational grid aligned with coastal boundaries to enhance spatial referencing in data, facilitating bidirectional retrieval from catalogs and spatial perspectives. Lastly, the knowledge network describing the associations between coastal zone data and models must be constructed to guide the information integration. Considering the input-output relationships of data and models across different domains, the chain relationships between coastal-related data from various interfaces and the simulated analytical data resulting from model processing could be built.

Coastal model integration of the CZIM focuses on the limitations of traditional models or AI methods in elucidating the dynamic processes of integrated coastal systems. It can be carried out from the perspective of dynamic model integration, including optimizing local processes, revising characteristic criteria, establishing nested models, and constructing ensemble models in different stages of model calculation. It is significant to delineate mechanistic processes at different stages of the coastal zone system to construct the model integration framework. On this basis, causal chains could be formulated for models specific to different scenarios (e.g., typhoon assessment, estuarine water level increase). Inherent assumptions and parameter design in different computation stages should be adaptive in the foundational configurations. In the input-output process part, the input-output for different coastal models with coordination, nesting, and coupling approaches should be standardized to facilitate collaboration with different computing domains. In the results analysis, statistical and learning-based computational methods should be combined to synthesize a large volume of computational results, addressing inherent shortcomings in time, space, and accuracy.

The CZIM standardized knowledge system primarily aims to establish connections between coastal scientific knowledge and direct stakeholders in coastal management. It integrates global, regional, and indigenous knowledge to guide users at different knowledge levels in applying coastal information. Its core architecture comprises multi-domain knowledge induction, multi-scale knowledge integration, and knowledge guidance for diverse audiences. Multi-domain knowledge induction aims to combine nature and humanities knowledge to build connections between the two, which involves three categories of knowledge: natural, human, and association knowledge. Natural knowledge can be induced by establishing interactive relationships across different interfaces of coastal zones, the causality of natural events, and their transformation over time and space. For human management knowledge, standardized decision chains in various departments need to be developed by analyzing business flows across different departments, identifying associated patterns, summarizing responses, and analyzing chains. For association knowledge, the association network between human-nature knowledge and stakeholders in practical production activities should be established by establishing the collaborative community and rules to support the sustainable development of the coastal zone. Multi-scale knowledge integration aims to integrate coastal management policies at different scales from global to regional perspectives. It can be achieved through integrating the international environment and national policies, sustainable development goals and regional indigenous knowledge, and global change and regional resilience. It can support finding the optimal paths for regional coastal zones under systematic knowledge integration. Knowledge guidance for diverse audiences aims to eliminate the knowledge barriers in applying professional domain information through knowledge guidance for different audiences, addressing diverse information recipients, and utilizing techniques such as multimedia, intelligent question-answering systems, and knowledge graphics.

CZIM’s scenario-based interactive information system aims to create user-friendly, knowledge-guided, and 3D reality support coastal digital scene systems. The coastal zone requires scenario-based management for various purposes, including coastal infrastructure projects, port and maritime regulation, land-sea spatial planning, and comprehensive management scenarios such as typhoon disaster assessment or sustainable development management. Combining natural scenes and varied human management demands in the system construction necessitates addressing three key issues: foundational scene construction, multi-dimensional rendering of natural attributes, and interaction between user and system. The transformation from traditional 2D-oriented coastal digitization systems to 3D and multi-information fusion patterns is essential. This involves 3D rendering for entities and high-dimension representation for land and sea environmental factors, especially for marine and atmospheric environmental factors such as air and ocean components with a height dimension. CZIM should enable the development of more intelligent and convenient interaction modes. For instance, it combines LLM development with a question-and-answer (Q/A) interactive approach tailored for comprehensive coastal information integration. Utilizing LLM as the core, it interprets interactive answers, automates the invocation of data and models, and renders scene results. This simplifies intricate interaction logic and achieves an intelligent interactive coastal information system.

## 4 Challenges and future developments of CZIM

### 4.1 Scenario-based coastal data model

Coastal zones are characterized by various data application scenarios, such as tidal wetlands, estuaries, drainage lagoons, harbor transportation, and ecological evolution and resilience. CZIM provides a standardized and structured information and knowledge integration paradigm for these research and application scenarios. At the data governance level, CZIM’s data classification system, spatial variational grid, and data association system are vital in labeling data related to each scenario with spatial, domain, and application attributes. This data management system establishes a strong coupling between the research scenario and its related data, enabling efficient selection, replacement and merging of big earth data related to coastal zones on demand. It also simplifies the management and expression of data in spatial and non-spatial databases and visualization engines in digital twin systems. At the data association level, CZIM’s data are widely associated with different coastal sub-domain systems, each with its domain constraints. This association of domains and data offers the potential to merge them and construct more complex scenarios with controllable options for every detail.

At the data application level, there is still a need for a data model to support efficient data management and update of vital natural scenarios of the coastal zone. A standard data model requires stable observation sources, unique classification, and fixed storage methods. Coastal zone scenarios include complex spatial patterns, topography, and hydrodynamic processes and do not satisfy the general condition of the data model to some extent. Nevertheless, constructing the coastal zone scenario data model is still the foundation to support the realization of the coastal digital twin. Through the data model, data collection, governance, reconstruction and visualization can form a complete chain that achieves rapid end-to-end application. The standard specification of the data model can maximize the possibility of data community sharing, supporting not only researchers but also system developers and data users in the use of data across the different platforms to form a virtuous cycle of the coastal research ecology. This also means digital construction in coastal zones needs more in-depth cooperation with the different domains and international institutions.

### 4.2 Coastal model coordination for analysis services

Domain knowledge and data support are the core of developing coastal statistical, dynamic, or AI modeling. CZIM provides comprehensive capacity in data management for coastal modeling by classifying, spatially discretizing, and associating the multi-source coastal data. Such a management framework could complete the entire data application chain and provide an environment for coastal models to fuse data causally and spatially, especially for AI models, which are more critical and sensitive for big data fusion. Regarding coastal domain knowledge integration, there is still a large gap in research communities that analyze the sub-processes of coastal zone interactions from an integrated complex systems perspective. For model integration, the coastal processes at different scales and domains must be finely tuned, including the processes’ mechanism, mathematical description, model implementation, and inputs and outputs. The process coupling can only be rationalized by understanding its interaction process. Accordingly, the analysis dimension of the coastal multi-scale features should shift from a single field to a diversified one, and the connotation should be deconstructed from diverse domains and perspectives.

The computational model based on 3D virtual reality needs to be further developed. It’s valuable for decision-making scenarios such as coastal disaster prevention, spatial planning, and mitigation assessment. For example, the current risk-related assessment model (including explosibility, vulnerability, and stability) still operates in the two-dimensional planar coupling and computational mode on a large scale, and the original three-dimensional disaster-bearing bodies in the coastal zone are ignored (such as urban buildings, and coastal engineering). Three-dimensional territorial spatial planning is also urgently needed for the scientific utilization of coastal spatial resources. There is a need for the development of a 3D scenario-based risk assessment model, and the CZIM provides potential application opportunities.

### 4.3 Scenario-based intelligent interactions

The CZIM provides a standardized paradigm for aggregating coastal zone information, encompassing the standard integration and application of data, models, and knowledge. It could provide the foundation to enrich the practical connotations of digital twins in the coastal zone. The coastal zone digital twin is a systematic project from the underlying data logic to the top-level macro-architecture to realize the coastal data, model, and knowledge integration and guide the real and virtual coast reciprocal feedback. However, the pre-application processes of retrieving, reorganizing, transmitting, parsing, and rendering of information are generally redundant for users. Model invocation, result output, and data analysis are unfamiliar, especially for non-professionals. In this context, the LLM’s Q/A interactive mode offers a way out of this predicament. The LLM is a kind of intelligent model training that can understand and analyze the connotations of human language [96]. For example, it could interpret user queries and provide relevant data and model outputs. For the combined interactive needs of various coastal zone scenarios, information, and knowledge, only Q/A may not achieve intelligent interaction. By intervening in the responses of the LLM, the answer format can present standardized guidance on data, model applications, and knowledge. This is coupled with governance modules for data retrieval and reorganization operations, integrated modules for model invocation and analysis, and expression and manipulation modules for rendering and analyzing information. This integrated approach holds the potential to achieve seamless, intelligent interaction from questioning to obtaining results. While there is still a certain distance to realize the intelligent interactions in coastal zone information, it is foreseeable that with ongoing fine-tuning, embedding, and advancement of operational techniques for domain-specific LLM, the LLM tailored for coastal information interaction could become a crucial technology to support the application of digital twin coastal zones.

## 5 Conclusion

In this paper, a novel framework of CZIM is proposed to integrate the coastal heterogeneous information through standardized specifications and architectures to achieve integration, cooperation, and development across coastal multi-disciplinary knowledge, data, and models. A comprehensive review reveals that the current coastal zone digitization is still challenging in the integration of coastal knowledge and information and its application to support the coastal zone’s sustainable development. A collaborative architecture of the CZIM consisting of four modules of coastal-wide data governance, multi-domain model integration, standardized knowledge system, and scenario-based interactive systems is discussed in detail. The CZIM is ultimately used to improve coastal management capabilities in response to both internal and external forces, such as socioeconomic development, global warming, and rising sea levels. It also provides a valuable introduction to multi-disciplinary cooperation in coastal research and digital system construction.

In the future, our research will be guided by a deeper understanding of the characteristics and demands of information applications and knowledge integration in different coastal domains. We will focus on three key directions: Firstly, we aim to develop a scenario-based coastal zone data model for dynamic and sustainable analysis of coastal zone scenarios. Secondly, we plan to refine the model integration methods, explicitly focusing on coastal sustainable development assessment and decisions supported by real scenarios. Lastly, we will train the Coastal Zone Large Language Model and refine the Coastal Zone Intelligent Interaction methodology. These future directions underscore our commitment to continuous improvement and the potential for further development of the proposed model.

## Declaration of competing interest

The authors declare that they have no conflicts of interest in this work.

## Footnotes

1 The mapping 4D products including the Digital Elevation Model (DEM), Digital Surface Model (DSM), Digital Orthophoto Map (DOM), and Digital Line Graphic (DLG).
